# Exploring the dynamics of snoring in relation to sleep stages: Implications for gender differences, sleep position, and upper airway collapsibility

**DOI:** 10.1371/journal.pone.0295232

**Published:** 2024-01-31

**Authors:** Masaaki Suzuki, Yuichi Kawai, Yoshihiro Funayama

**Affiliations:** 1 Dept. of Otorhinolaryngology, Teikyo University Chiba Medical Center, Chiba, Japan; 2 Division of Sleep Medicine, Kaname Clinic, Tokyo, Japan; University of Verona: Universita degli Studi di Verona, ITALY

## Abstract

**Objective:**

The purpose of this study was to determine the sleep stage during which isolated snoring occurs in children and adults, and to analyze changes after treatment of obstructive sleep apnea (OSA).

**Methods:**

This retrospective study examined duration of snoring time and respiratory events during each sleep stage in adults and children who underwent polysomnography, had an apnea-hypopnea index (AHI) < 15/h and had snoring time ≥ 10% of total sleep time. Changes in duration of snoring time were also examined in adult patients after treatment with an oral appliance (OA).

**Results:**

Snoring time was shown to be predominant during N3 and N2 sleep and less dominant during REM sleep in both children (*n* = 47) and adults (*n* = 93). These results were seen even in children with REM dependency. The percentage of snoring time during N3 sleep was more pronounced in women than in men among young adult patients aged < 40 years but was not significantly different between men and women overall. There were no significant differences in the percentage of snoring time in each sleep stage between young women with mild OSA and non-OSA. In children, there were no significant differences between boys and girls in the percentage of snoring time in any sleep stage. The percentage of snoring time during N3 was significantly higher sleep in the non-supine position than in the supine position in children, whereas no significant differences were noted between the supine and non-supine positions in any sleep stage in adults. OA treatment for adult patients (*n* = 20) significantly increased the percentage of snoring time during N3 sleep, although it significantly decreased AHI, total snoring time, and snoring time during N1 sleep and REM sleep.

**Conclusions:**

Snoring presented exclusively during the N3 sleep stage, especially in young women with mild OSA, and in children with OSA, especially in the non-supine position. Snoring time during N3 sleep increased during OA treatment for OSA.

## Introduction

Snoring is a respiratory sound originating in the upper airway during sleep that results from vibration of the pharyngeal wall and associated structures, which typically occurs during inspiration but can also occur during expiration [1]. Snoring can be classified into two main patterns: snoring associated with respiratory events, in which snoring occurs during or at the end of respiratory events such as apnea, hypopnea, or respiratory effort-related arousal during sleep; and snoring independent of respiratory events during sleep, referred to as isolated, primary, or simple snoring [2]. It is well known that respiratory events are often more pronounced during stage N2 sleep and rapid eye movement (REM) sleep, and it is speculated that differences exist between the sleep stage in which respiratory events appear and those in which isolated snoring appears. However, few studies have investigated snoring independent of respiratory events. Those studies using polysomnography (PSG) have revealed a higher snoring index during slow wave sleep compared with other sleep stages and a higher snoring index in all sleep stages in men without obstructive sleep apnea (OSA) compared with women without OSA [3] as well as in adult men and women with a mean apnea-hypopnea index (AHI) of 22 ± 15.9/h and 28 ± 17/h, respectively, indicating the inclusion of participants with moderate to severe OSA [4].

Against this background, this study sought to determine at which sleep stage isolated snoring occurs in children and adults, especially those with mild OSA and categorized by age group, and to analyze changes after OA treatment in adult patients.

## Methods

This retrospective study comprised two parts. First, we analyzed data on duration of snoring time and respiratory events during each sleep stage from adults (age > 18 years) with AHI ≥ 5/h but < 15/h and children with AHI ≥ 1/h, both with ≥ 10% snoring time of total sleep time (TST). Second, among adult patients whose AHI and percentage of sleep time in N3 sleep showed improvement after treatment with an oral appliance (OA), we analyzed changes in duration of snoring time in TST during each sleep stage.

### Polysomnography

All participants underwent standard type 1 in-laboratory overnight PSG (Alice 6; Philips Respironics, Pittsburgh, PA) for diagnosis and for determining OA-set outcome measurements. In brief, electroencephalography (C4/A1, C3/A2), electrooculography, submental surface electromyography, and electrocardiography using surface electrodes were performed. Nasal air flow was measured with pressure and thermistor sensors at the nostrils, and respiratory movements of the rib cage and abdomen were measured by inductive plethysmography. Percutaneous arterial oxygen saturation using finger pulse oximetry was also measured. Body position was measured using a position sensor, with a mercury switch attached to the anterior chest wall on the median line. A microphone was placed on the neck to detect snoring (dB > 20). Snoring events were scored manually, and then the PSG system automatically calculated total duration of snoring time. PSG measurements were scored manually by registered PSG technologists. Percentage of sleep time in TST (%sleep time), percentage of duration of respiratory events, and percentage of snoring time were calculated for each sleep stage. Adult patients with an obstructive AHI score ≥ 5/h but < 15/h were considered to have mild OSA, with an obstructive AHI score < 5/h indicating no OSA [5]. REM sleep dependency was identified when the value of AHI during REM sleep divided by the AHI during non-REM sleep was ≥ 2.

### Treatment with an oral appliance

OAs were made of self-cured acrylic resin as described in our previous study [6]. Each OA was custom-made and adjusted individually by a dentist, with the participant instructed on its use. After taking impressions of the maxillary and mandibular dental arches, maxillary and mandibular portions were prepared separately to maintain the mandible in an anteriorly protruding position. If the patient complained of any discomfort in the temporomandibular joints or muscles after using the OA, the mandibular portion was set at a less-protruding position. This adjustment was repeated until the patient could wear the OA continuously without problem before the device-set PSG study was conducted. No OA titrations were performed. Device-set PSGs were basically performed 8 weeks after final device instruction.

### Ethical consideration

The ethics committee at our institute approved this study (Approval No. Teirin 19–070) and written informed consent was obtained from all participants. The institutional review board waived the requirement to obtain consent from parents or guardians of minors in this study. Patients consented to the use of their PSG data for the study at the time of their first visit to our outpatient clinic. The PSG examinations and the oral appliance described in this study was implemented as standard-of-care. Patients who declined to take part in the study received the same care as the patients who agreed to take part in the study. Data were collected from 2018 to 2022. The authors conducted this study in December 2022. The source of data was a sleep center affiliated with a university hospital. The authors did not have access to information that could identify individual participants during or after data collection.

### Statistics

Descriptive statistics for all variables are presented as the mean ± standard deviation. Paired and unpaired comparisons were evaluated by the Wilcoxon signed rank test and Mann–Whitney U test, respectively. Changes in values among three sleep parameters were evaluated by one-way analysis of variance. Bonferroni correction was applied for multiple comparisons (post hoc tests). Statistical significance was set at *p* < 0.05. All statistical analyses were performed using the IBM Statistical Package for Social Science (ver. 26; IBM Inc., Armonk, NY).

## Results

**Fig 1:**
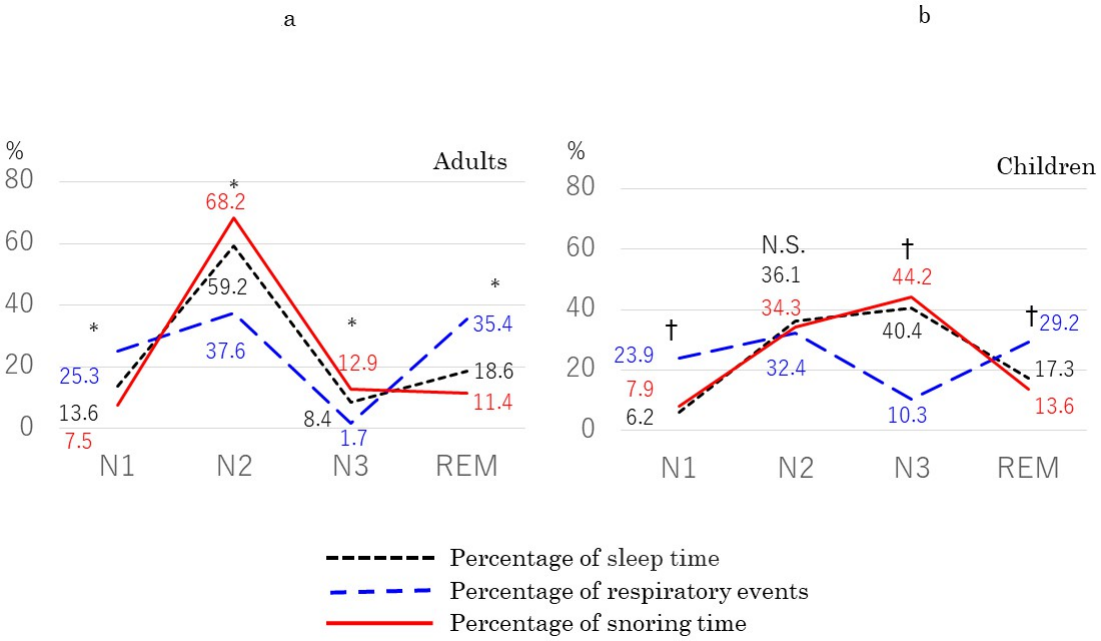
Distribution of percentage of sleep time, respiratory events, and snoring time in each sleep stage in (a) adults with mild OSA (*n* = 93) and (b) children with OSA (age 3–12 years, *n* = 47). Black short-dashed line: percentage of sleep time. Blue long-dashed line: percentage of respiratory events. Red solid line: percentage of snoring time. *Significant difference between the three parameters. †Significant differences between percentage of respiratory events and the other two parameters. N.S.: not significant.

In the first part of this study, we analyzed data from 93 adults with mild OSA (mean age, 38.7 ± 14.3 years; mean AHI, 10.2 ± 3.1/h; mean body mass index [BMI], 23.3 ± 3.9 kg/m^2^) and 47 children with OSA (mean age: 7.5 ± 2.5, range 3–12; mean AHI: 5.0 ± 4.0/h). In the adult patients with mild OSA (Fig 1a), %sleep time was 13.6% ± 5.7% during N1 sleep, 59.2% ± 6.8% during N2, 8.4% ± 6.4% during N3, and 18.6% ± 6.0% during REM sleep, and percentage of respiratory events was 25.3% ± 15.3% during N1 sleep, 37.6% ± 16.5% during N2, 1.7% ± 4.7% during N3, and 35.4% ± 21.1% during REM sleep. Percentage of snoring time in TST was 7.5% ± 6.0 during N1 sleep, 68.2% ± 15.2 during N2, 12.9% ± 13.2 during N3, and 11.4% ± 9.5 during REM sleep. There were significant differences among the three parameters in every stage. In children with OSA (Fig 1b), %sleep time was 6.2% ± 3.2% during N1 sleep, 36.1% ± 11.3% during N2, 40.4% ± 13.7% during N3, and 17.3% ± 5.3% during REM sleep, and percentage of respiratory events was 23.9% ± 19.3 during N1 sleep, 32.4% ± 23.4% during N2, 10.3% ± 13.3% during N3, and 29.2% ± 23.0% during REM sleep. Percentage of snoring time in TST was distributed 7.9% ± 5.5 during N1, 34.3% ± 17.1 during N2, 44.2% ± 21.9 during N3, and 13.65 ± 12.4 during REM sleep. In ad hoc analyses, we found no significant difference between %sleep time and percentage of snoring time in any sleep stage, whereas there were significant differences between these two parameters and percentage of respiratory events in N1, N3, and REM sleep.

**Fig 2:**
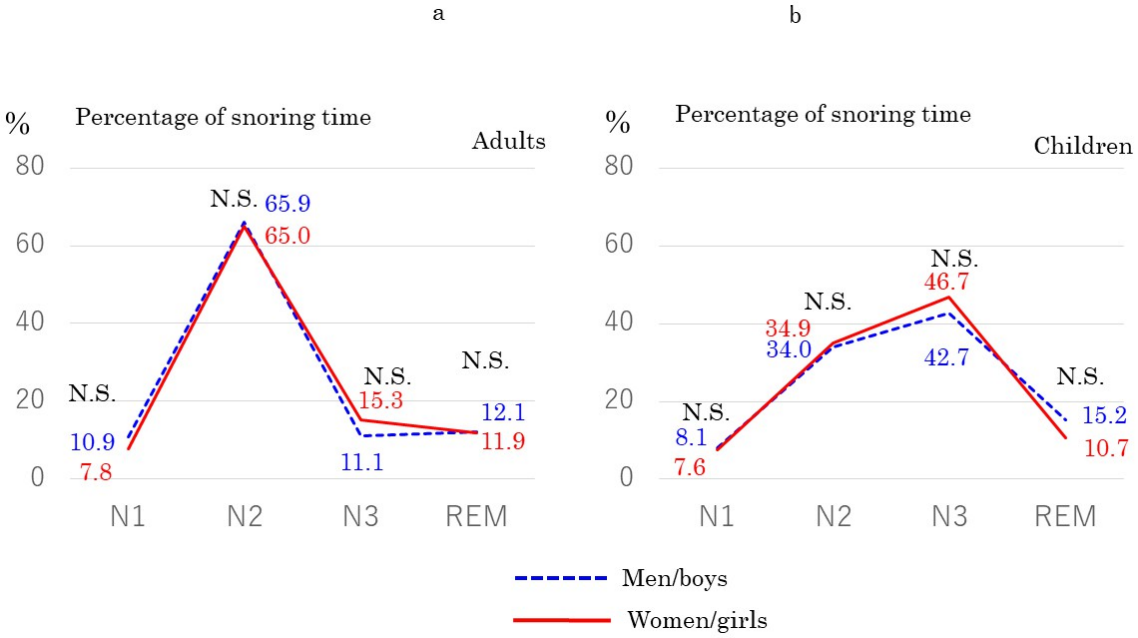
Comparison of percentage of snoring time in each sleep stage between (a) men (*n* = 34) and women (*n* = 59) with mild OSA and (b) between boys (*n* = 30) and girls (*n* = 17) with OSA. Blue dashed line: men or boys: Red solid line: women or girls. N.S.: not significant.

**Fig 3:**
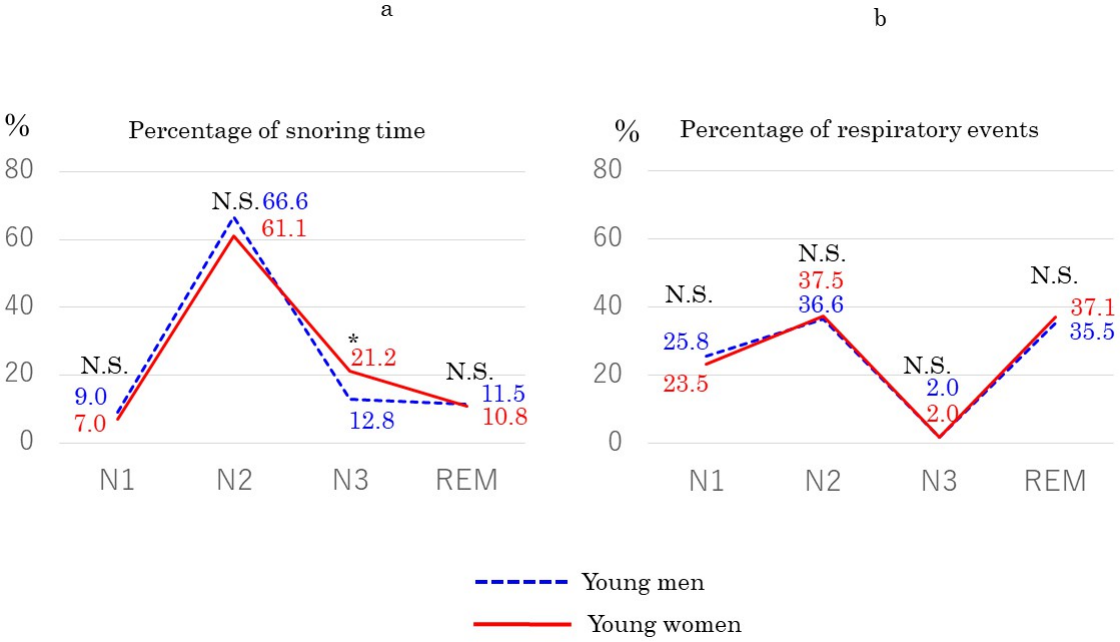
Comparison of percentage of (a) snoring time and (b) respiratory events in each sleep stage between young men (*n* = 29) and young women (*n* = 33) aged < 40 years with mild OSA. Blue dashed line: men. Red solid line: women. N.S.: not significant. *Significant difference.

In a comparison between all men (*n* = 34; mean AHI: 10.7/h ± 2.6; mean age: 34.3 ± 10.4; mean BMI: 24.3 kg/m^2^ ± 3.2) and all women (*n* = 59; mean AHI: 9.8/h ± 3.4; age: 41.2 ± 15.6; BMI: 22.4 kg/m^2^ ± 4.3), we found no significant differences in AHI (*p* = 0.88) or age (*p* = 0.08) but significant differences in BMI (*p* = 0.001). We also found no significant difference in percentage of snoring time in any sleep stage between them (Fig 2a). However, among adult patients aged < 40 years, percentage of snoring time was significantly higher in women (*n* = 33) than in men (*n* = 29) during stage N3 sleep (*p* = 0.03), whereas we found no significant differences in percentage of respiratory events (Fig 3). In addition, we compared the percentage of snoring time between women aged < 40 years with mild OSA and non-OSA (*n* = 15, mean age: 31.7 ± 9.6, mean AHI: 2.4/h ± 1.4, mean BMI 24.5 kg/m^2^ ± 4.5). There were no significant differences in the percentage of snoring time in any sleep stage. In children, we found no significant differences in AHI (*p* = 0.15) or age (*p* = 0.45) between boys (*n* = 30; mean AHI, 4.3 ± 3.8/h; mean age, 7.8 ± 2.7 years) and girls (*n* = 17; mean AHI, 6.1 ± 4.2/h; mean age, 7.2 ± 2.5 years) and no significant difference in percentage of snoring time in any sleep stage (Fig 2b).

**Fig 4:**
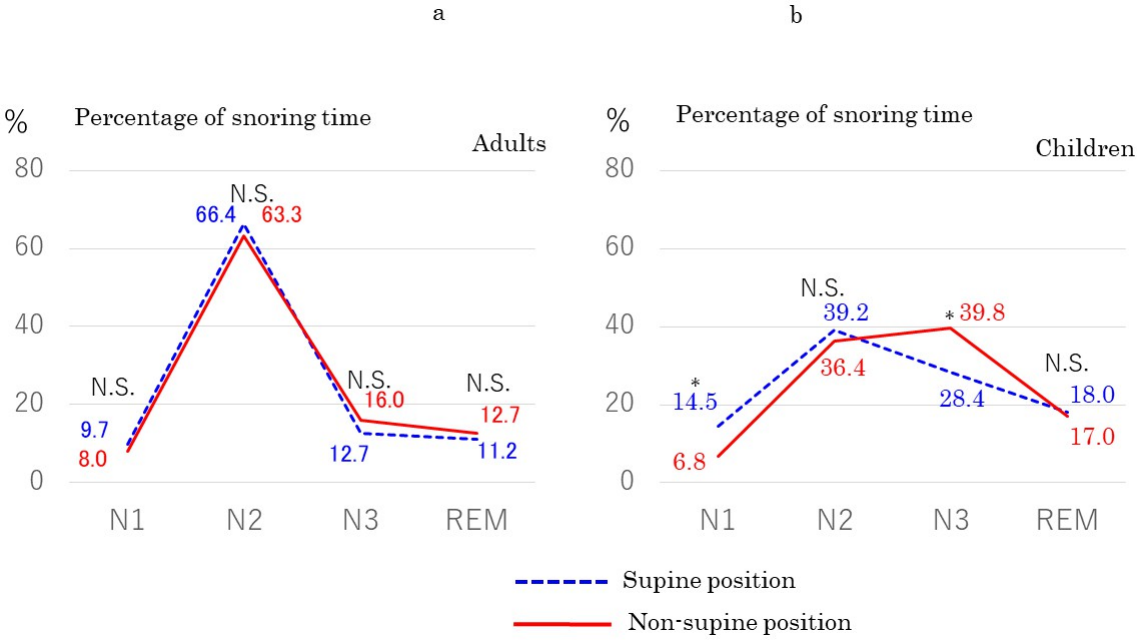
Comparison of percentage of snoring time in each sleep stage between the supine and non-supine positions in (a) adults with mild OSA (*n* = 93) and (b) children with OSA (*n* = 47). Blue dashed line: supine position. Red solid line: non-supine position. N.S.: not significant. *Significant difference.

We compared the percentage of snoring time between the supine and non-supine (i.e., left and right lateral, and prone) positions. In adult patients (*n* = 93), there were no significant differences in the percentage of snoring time between the supine and non-supine positions in any sleep stage (Fig 4a). However, in children (*n* = 47), the percentage of snoring time in the non-supine position was significantly higher during N3 sleep (*p* = 0.046) and significantly lower during N1 sleep (*p* = 0.043) compared with the supine position (Fig 4b). A similar tendency was also seen in adult women aged < 40 years (n = 33) but the difference was not significant (no figure).

**Fig 5:**
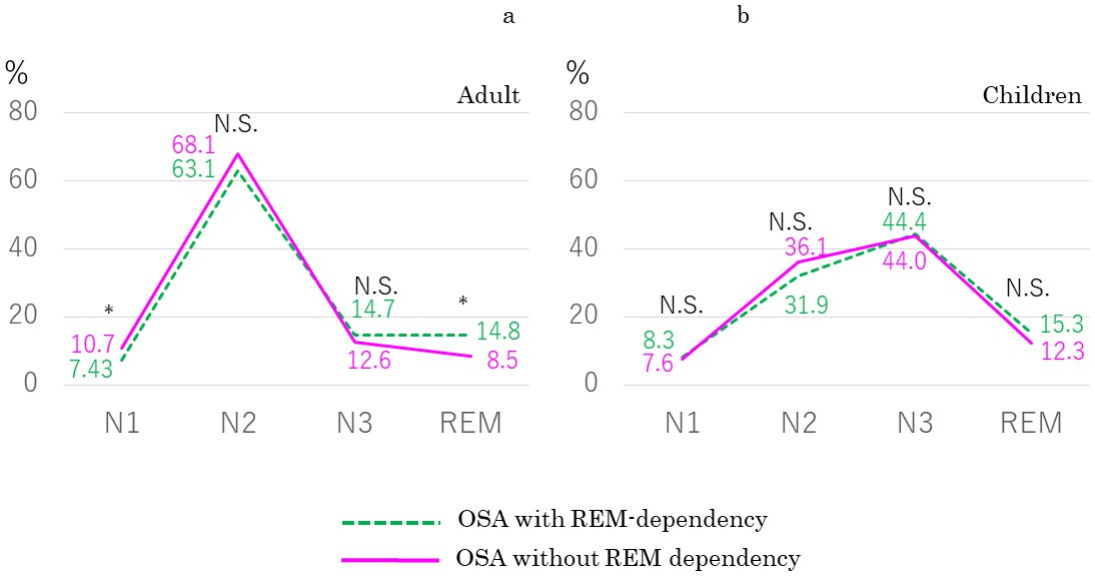
Comparison of percentage of snoring time in each sleep stage (a) between adult patients with (*n* = 51) and without REM-dependency (*n* = 42) and (b) between children with (*n* = 20) and without REM-dependency (*n* = 27). REM-dependency: REM AHI/non-REM AHI ≥ 2. Green dashed line: patients with REM-dependency. Pink solid line: patients without REM-dependency.

In a comparison between adult patients with REM sleep dependency (*n* = 51; mean AHI, 9.8 ± 3.1/h; age, 40.4 ± 13.9 years; BMI, 23.6 ± 4.1 kg/m^2^) and those without (*n* = 42; mean AHI, 10.6 ± 3.1/h; age, 36.6 ± 14.7 years; BMI, 23.0 ± 3.7 kg/m^2^), we found no significant difference in AHI (*p* = 0.26), age (*p* = 0.09), or BMI (*p* = 0.55). Percentage of snoring time in adult patients with REM sleep dependency was significantly higher during REM sleep (*p* = 0.01) and lower during N1 sleep (*p* = 0.01) than in adult patients without REM sleep dependency (Fig 5a), although these percentages were much lower than for respiratory events during each sleep stage. When we compared children with REM sleep dependency (*n* = 20; mean AHI, 4.6 ± 4.0/h; mean age, 7.7 ± 2.7 years) and those without (*n* = 27; mean AHI, 5.2 ± 4.1/h; mean age, 7.2 ± 2.3 years), we also found no significant differences in AHI (*p* = 0.9) or age (*p* = 0.45), but also found no significant difference in percentage of snoring time in any sleep stage between the two groups (Fig 5b).

**Fig 6:**
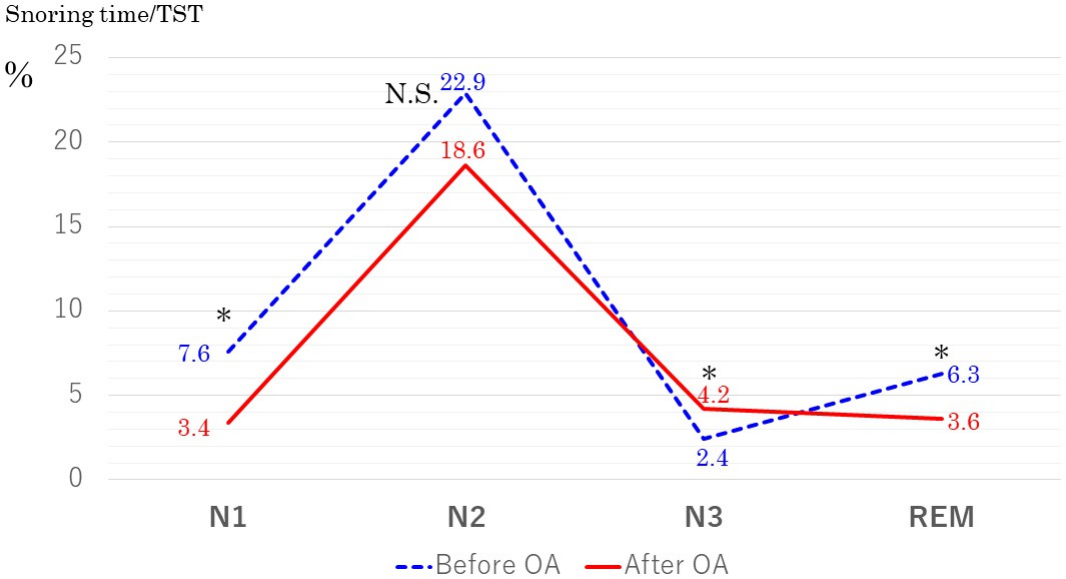
Comparison of percentage of snoring time in total sleep time (TST) in each sleep stage before and after oral appliance (OA) treatment. Blue dashed line: patients before OA treatment. Red solid line: patients after OA treatment.

In the second part of this study, we examined the effects of OA treatment on snoring in 20 adult patients (mean age, 47.9 ± 13.6 years; mean AHI, 16.2 ± 4.9/h; mean BMI, 24.9 ± 4.7 kg/m^2^). OA treatment significantly decreased AHI from 16.2 ± 4.9/h to 10.1 ± 4.9/h (*p* < 0.001) and increased the percentage of N3 sleep in TST from 4.4% ± 4.9% to 9.0% ± 4.6% (*p* < 0.001). When we compared snoring time in TST between before and after OA treatment, there was a significant increase in percentage of snoring time during N3 sleep (*p* = 0.028) and significant decreases in total snoring time (from 41.8% ± 14.1% to 30.3% ± 18.8%, *p* = 0.05) and in snoring time during N1 (*p* = 0.002) and REM (*p* = 0.007) during OA treatment (Fig 6).

## Discussion

This study obtained several novel findings. First, snoring time was shown to be predominant during N3 and N2 sleep and less dominant during REM sleep in adult patients with mild OSA and children with OSA. This tendency was seen even in children with REM dependency. Second, percentage of snoring time during N3 sleep was more pronounced in women than in men among adult patients aged < 40 years with mild OSA, while there were no significant differences between men and women overall. There were no significant differences in the percentage of snoring time in any sleep stage between young women with mild OSA and non-OSA. Third, the percentage of snoring time during N3 sleep was significantly higher in the non-supine position than in the supine position in children. Fourth, in adult patients whose AHI and percentage of N3 sleep stage were improved with OA treatment, snoring time during N3 sleep increased significantly during OA treatment, but total snoring time and snoring time during N1 and REM sleep were significantly improved. To our knowledge, this study is the first to use PSG to analyze the stage of sleep at which isolated snoring occurs in adult patients with mild OSA by age group, and in children with OSA, and to analyze changes after OA treatment for OSA.

### Do respiratory events always develop from snoring events?

Snoring is considered a prelude to a respiratory event during sleep. Respiratory arousals that accompany respiratory events shift deep sleep to shallow sleep, whereas snoring events without arousals do not alter sleep stage. It can be assumed that deep sleep can occur only when there are few or no respiratory events. Thus, snoring presents predominantly during deep sleep while respiratory events present during shallow sleep. Snoring events are thought to develop into respiratory events during non-REM sleep. In contrast, in this study, snoring was found to be less dominant and respiratory events predominant during REM sleep. REM sleep diminishes genioglossus activity secondary to the cholinergic mediated inhibition of hypoglossal motor output; therefore, REM sleep is simply a period of increased vulnerability of the upper airway [7]. In addition, thoracic respiration is reduced and abdominal respiration via the phrenic nerve becomes dominant during REM sleep. On the other hand, consolidation of recognition memory and modulation of emotional reactivity are most crucial during REM sleep. During REM sleep, strong reactivation of brain areas implicated in daytime memory function and overnight reprocessing of emotional memories would be supported [8]. As protection of the brain might be most important during REM sleep, breathing that becomes unstable in REM sleep shifts to a respiratory event without going through snoring.

Collapsibility of the pharyngeal wall may be highest during REM sleep, followed by N1, N2, and N3 sleep. Snoring may require some degree of pharyngeal wall retention; thus, although snoring is likely to occur during N3 sleep, respiratory events are not. The hypothesis that a snoring event develops into a hypopnea/apnea event could be true during non-REM sleep but not always true during REM sleep.

### Sex differences in snoring during non-REM sleep

This study revealed that snoring during slow wave sleep was more pronounced in women than in men among younger adults aged < 40 years with mild OSA. Upper airway collapsibility, which is determined by measuring critical closing pressure (i.e., positive or negative pressure accompanying airway collapse), is reported to be greater in men than in women after controlling for BMI, age, and apnea severity [9]. Likewise, healthy men have exhibited a significantly higher increase in pharyngeal resistance in response to inspiratory loading during non-REM sleep compared to women despite a similar ventilatory response to the load [10]. In addition, central chemoreflex sensitivity is greater in males than in females, indicating that ventilator response to carbon dioxide and hypoxemia is increased in men compared to women [11]. The effect of estrogen and/or progesterone on the chemical control of breathing might play a role in the prevalence of sleep-disordered breathing [11]. These sex differences could account for the lower incidence of respiratory events and higher incidence of isolated snoring during N3 sleep in women compared to men. Isolated snoring without arousal usually does not shift deep sleep to shallow sleep as mentioned above. The reduced airway resistance in women due to increased tonicity of upper airway muscles is positively correlated with progesterone levels. This might partially explain the differences between younger and older females [12].

### Can patients always decrease snoring by sleeping in a non-supine position?

Positional dependency is a common phenotype. It is reported that the supine position impairs sleep quality primarily through increased respiratory events and sleep fragmentation [13]. The primary pathophysiology of positional dependence is regarded as an anatomical issue [14]. Differences in the distribution of soft tissue surrounding the pharyngeal airway suggest that tissue pressure might vary axially depending on the direction of gravity relative to the patient’s position [14]. One study reported that body posture significantly influenced the pharyngeal critical closing pressure, with mean values of 0.3 ± 0.5 cm H_2_O for lateral body posture and 2.5 ± 0.5 cm H_2_O for supine body posture, which accounts for less pharyngeal collapsibility in the lateral position compared with the supine position [15]. Pharyngeal airway patency in children also has been reported to improve in the non-supine position compared with the supine position [16]. Respiratory events would be more likely to occur when the pharyngeal wall is vulnerable to collapse; however, snoring might be more likely to occur when retention of the pharyngeal wall is somewhat sustained. Our results suggested that retention of the pharyngeal wall during N3 is more sustained in the non-supine position compared with the supine position, especially in children. A lateral position would decrease respiratory events but might increase snoring during N3 sleep, and thus the hypothesis that patients can decrease snoring by sleeping in a non-supine position may not always be true during N3 sleep, especially in children.

### Is residual snoring during treatment caused only by lack of pharyngeal patency?

Experimental studies have shown that the pharyngeal critical closing pressure progressively increases from negative levels to positive levels as the severity of OSA increases, indicating the mechanism underlying residual snoring after treatment is due to lack of pharyngeal opening [17]. However, our results suggest that changes in N3 sleep reflect residual snoring during OA treatment. Residual snoring during or after treatment can be affected not only by lack of pharyngeal opening but also by changes in sleep stages. Therefore, one reason CPAP users snore during treatment is insufficient pressure, but another reason could be increased deep sleep.

### Study limitations

In this study, we analyzed only duration of snoring time and not sound pressure level as measured in decibels. In research using advanced technology, sound pressure can be measured with high quality visual resolution [18]. In addition, sound analysis of snoring has recently been expanded by applying machine learning methods to PSG measurements [19], while electrophysiological signal recordings with PSG revealed the effects of snoring sounds on electroencephalography and electrocardiography signals [20]. Sensory and digital devices developed with computer-based learning methods have made it possible to diagnose and treat snoring and OSA [21]. Analyses using these advanced technologies might highlight additional aspects of snoring.

Second, the relationship between snoring and complications such as hypertension has not been evaluated. Several previous researchers have shown an independent association between snoring sound intensity and daytime blood pressure in patients with OSA and even in those without OSA [22–25]. Besides snoring associated with respiratory events, exposure to vibratory stimuli caused by isolated snoring can damage the endothelial cells in arterial walls, which may trigger an inflammatory cascade leading to carotid atherosclerosis [26–28].

In conclusion, snoring time was predominant during the N3 and N2 sleep stage, especially in children with OSA and in young women aged < 40 years with mild OSA. Snoring time during N3 sleep increased during OA treatment in adult patients when OA treatment improved their AHI and percentage of N3 sleep. The hypothesis that a hypopnea/apnea event develops from a snoring event could be true during non-REM sleep but not always during REM sleep. The hypothesis that patients can decrease snoring by sleeping in a non-supine position may not always be true during N3 sleep, especially in children. Residual snoring during or after treatment could be affected not only by lack of pharyngeal opening but also by changes in sleep stages.

## Supporting information

S1 Data (XLS)

S1 Checklist STROBE statement—Checklist of items that should be included in reports of observational studies. (DOCX)
