# Supplementary material for: Exploring the dynamics of snoring in relation to sleep stages: Implications for gender differences, sleep position, and upper airway collapsibility
Source: PLoS One. 2024 Jan 31;19(1):e0295232. doi: 10.1371/journal.pone.0295232 (PMC10830031; doi:10.1371/journal.pone.0295232)
Supplement: Attachment — Submitted filename: Response to Reviewers PLOS ONE.docx [file pone.0295232.s003.docx]

Reviewer #1:

We would like to thank the reviewer for the latest review and we have answered each of the points below. The paper has been checked by a native English-speaking medical editor. We hope that this more substantial reworking of the manuscript is satisfactory.

Among the events that disrupt sleep health in the world, snoring and sleep breathing disorders are common and well known. I find the research review necessary and important. My suggestions and comments to the authors explaining the "relationship between snoring and sleep stages" in adults and children in their articles are as follows:
Comment 1: Presenting the "percentage of snoring time in TST" values in a table makes it easy to see each value (for the Results section).

Thank you for pointing this out. For clarity, all numbers are presented in numerical form. See figures.
Comment 2: For the discussion section, innovative diagnosis and treatments should be presented, apart from the classical treatment of snoring Treatment with an oral appliance. Recently, snoring sound analyzes have been performed meticulously and successfully with machine learning methods applied to polysomnographic measurements (1). In this way, the effects of snoring sound on brain waves (ElectroEncephaloGram, EEG (2), respiration and heart (ElectroCardioGram)) are revealed by examining electrophysiological signal recordings in PSG. Again, by developing sensory and digital devices with computer-based learning methods, diagnosis and treatment of snoring and sleep breathing disorders is done (3).
Thank you for pointing this out. We have added this limitation with references to the Discussion.

Comment 3: For the study limitations section: The position of the snorer during sleep in NREM and REM sleep processes is very important in terms of reducing and treating snoring. Correct position of the person during sleep in bed (left or right side, less on his back); It improves NREM and REM sleep durations (3, 4). The results of this study do not provide information about the relationship between snoring and its position, especially in snoring women under the age of 40 and children aged 7 years.
Thank you very much for pointing this out. We compared the percentage of snoring time between the supine and non-supine (i.e., left and right lateral, and prone) positions.

In adult patients (*n* = 93), there were no significant differences in the percentage of snoring time between the supine and non-supine positions in any sleep stage (Fig. 4a). However, in children (*n* = 47), the percentage of snoring time in the non-supine position was significantly higher during N3 sleep (*p* = 0.046) and significantly lower during N1 sleep (*p* = 0.043) compared with the supine position (Fig. 4b). A similar tendency was also seen in adult women aged < 40 years (n = 33) but the difference was not significant.

We have added these findings to the Results section and figures, and we have revised the discussion accordingly.

Reviewer #2:

We would like to thank the reviewer for the latest review and we have answered each of the points below. The paper has been checked by a native English-speaking medical editor. We hope that this more substantial reworking of the manuscript is satisfactory.

A manuscript with important findings regarding NREM slow-wave sleep impact on OSA. One important key factor in any respiratory event (including snoring) in sleep apnea is body position. This manuscript does not include any information about body position. Adding this information can affect results and conclusions, therefore must be considered.

Thank you very much for pointing this out. We compared the percentage of snoring time between the supine and non-supine (i.e., left and right lateral, and prone) positions.

In adult patients (*n* = 93), there were no significant differences in the percentage of snoring time between the supine and non-supine positions in any sleep stage (Fig. 4a). However, in children (*n* = 47), the percentage of snoring time in the non-supine position was significantly higher during N3 sleep (*p* = 0.046) and significantly lower during N1 sleep (*p* = 0.043) compared with the supine position (Fig. 4b). A similar tendency was also seen in adult women aged < 40 years (n = 33) but the difference was not significant.

We have added these findings to the Results section and figures, and we have revised the discussion accordingly.
