# Supplementary material for: Exploring the dynamics of snoring in relation to sleep stages: Implications for gender differences, sleep position, and upper airway collapsibility
Source: PLoS One. 2024 Jan 31;19(1):e0295232. doi: 10.1371/journal.pone.0295232 (PMC10830031; doi:10.1371/journal.pone.0295232)
Supplement: Attachment — Submitted filename: renamed_359ed.docx [file pone.0295232.s004.docx]

We would like to thank the reviewer for the latest review and we have answered each of the points below. We hope that this more substantial reworking of the manuscript is satisfactory.

Additional Editor Comments
This study appears to address several issues related to snoring, sleep stages, and obstructive sleep apnea (OSA).

The study's title mentions "isolated snoring," but much of the content focuses on snoring in patients with OSA and during different sleep stages. This discrepancy can confuse readers and may not fully reflect the actual scope of the study. the title could be more accurate and representative. For instance, "Exploring the Dynamics of Snoring in Relation to Sleep Stages and Obstructive Sleep Apnea: Implications for Gender Differences and Upper Airway Collapsibility" might be a more suitable option. However, this is not strictly necessary..

Thank you for your wonderful suggestion. We agree and have changed the title to “Exploring the Dynamics of Snoring in Relation to Sleep Stages: Implications for Gender Differences, Sleep Position, and Upper Airway Collapsibility”. Since we analyzed the percentage of snoring time of non-OSA women, we do not want to include the word "OSA" in the title.

The study highlights that snoring is more predominant during N3 and N2 sleep stages and less present during REM sleep. These findings appear to align with some of the existing knowledge about sleep physiology and snoring. However, the discussion in the first passage and the description of the study results need to be more accurately presented. In the discussion, it is stated that snoring is predominant during deep sleep (N3) and less present during REM sleep, while the study suggests that snoring(respiratory events?) was less predominant during N3 and N2 sleep stages and more present during REM sleep. However, the discussion seems to contradict these results, creating confusion.
Thank you for pointing this out. Words and sentences were deleted and added to maintain consistency in the Discussion section.
