# Supplementary material for: Exploring the dynamics of snoring in relation to sleep stages: Implications for gender differences, sleep position, and upper airway collapsibility
Source: PLoS One. 2024 Jan 31;19(1):e0295232. doi: 10.1371/journal.pone.0295232 (PMC10830031; doi:10.1371/journal.pone.0295232)
Supplement: Peer review history

# Peer review: Decision Letter 0

**DOI:** 10.1371/journal.pone.0295232.r001

**Authors:** Marco Zaffanello

25 May 2023

PONE-D-23-01986During which sleep stages does isolated snoring occur in children and adults?PLOS ONE

Dear Dr. Suzuki,

Thank you for submitting your manuscript to PLOS ONE. After careful consideration, we feel that it has merit but does not fully meet PLOS ONE’s publication criteria as it currently stands. Therefore, we invite you to submit a revised version of the manuscript that addresses the points raised during the review process.

Please submit your revised manuscript by Jul 09 2023 11:59PM. If you will need more time than this to complete your revisions, please reply to this message or contact the journal office at plosone@plos.org. When you're ready to submit your revision, log on to https://www.editorialmanager.com/pone/ and select the 'Submissions Needing Revision' folder to locate your manuscript file.

Please include the following items when submitting your revised manuscript:

- A rebuttal letter that responds to each point raised by the academic editor and reviewer(s). You should upload this letter as a separate file labeled 'Response to Reviewers'.
- A marked-up copy of your manuscript that highlights changes made to the original version. You should upload this as a separate file labeled 'Revised Manuscript with Track Changes'.
- An unmarked version of your revised paper without tracked changes. You should upload this as a separate file labeled 'Manuscript'.

If you would like to make changes to your financial disclosure, please include your updated statement in your cover letter. Guidelines for resubmitting your figure files are available below the reviewer comments at the end of this letter.

If applicable, we recommend that you deposit your laboratory protocols in protocols.io to enhance the reproducibility of your results. Protocols.io assigns your protocol its own identifier (DOI) so that it can be cited independently in the future. For instructions see: https://journals.plos.org/plosone/s/submission-guidelines#loc-laboratory-protocols. Additionally, PLOS ONE offers an option for publishing peer-reviewed Lab Protocol articles, which describe protocols hosted on protocols.io. Read more information on sharing protocols at https://plos.org/protocols?utm_medium=editorial-email&utm_source=authorletters&utm_campaign=protocols.

We look forward to receiving your revised manuscript.

Kind regards,

Marco Zaffanello

Academic Editor

PLOS ONE

Journal Requirements:

When submitting your revision, we need you to address these additional requirements.

1. Please ensure that your manuscript meets PLOS ONE's style requirements, including those for file naming. The PLOS ONE style templates can be found at

https://journals.plos.org/plosone/s/file?id=wjVg/PLOSOne_formatting_sample_main_body.pdf and

https://journals.plos.org/plosone/s/file?id=ba62/PLOSOne_formatting_sample_title_authors_affiliations.pdf.

2. You indicated that you had ethical approval for your study. In your Methods section, please ensure you have also stated whether you obtained consent from parents or guardians of the minors included in the study or whether the research ethics committee or IRB specifically waived the need for their consent.

3. In your Data Availability statement, you have not specified where the minimal data set underlying the results described in your manuscript can be found. PLOS defines a study's minimal data set as the underlying data used to reach the conclusions drawn in the manuscript and any additional data required to replicate the reported study findings in their entirety. All PLOS journals require that the minimal data set be made fully available. For more information about our data policy, please see http://journals.plos.org/plosone/s/data-availability.

"Upon re-submitting your revised manuscript, please upload your study’s minimal underlying data set as either Supporting Information files or to a stable, public repository and include the relevant URLs, DOIs, or accession numbers within your revised cover letter. For a list of acceptable repositories, please see http://journals.plos.org/plosone/s/data-availability#loc-recommended-repositories. Any potentially identifying patient information must be fully anonymized.

Important: If there are ethical or legal restrictions to sharing your data publicly, please explain these restrictions in detail. Please see our guidelines for more information on what we consider unacceptable restrictions to publicly sharing data: http://journals.plos.org/plosone/s/data-availability#loc-unacceptable-data-access-restrictions. Note that it is not acceptable for the authors to be the sole named individuals responsible for ensuring data access.

We will update your Data Availability statement to reflect the information you provide in your cover letter.

Additional Editor Comments (if provided):

Dear Authors,

We would like to congratulate you on your research review focusing on snoring and sleep breathing disorders, which has received valuable feedback from the two reviewers. It is evident that your work addresses a common and significant issue that disrupts sleep health worldwide.

Reviewer 1 has suggested two specific improvements. Reviewer 2, who acknowledges the importance of your manuscript's findings. The decision is Major Revision

Considering the reviewers' expert opinions and constructive suggestions will greatly contribute to the overall quality and impact of your articles. We encourage you to carefully incorporate these revisions, ensuring that the revised manuscript effectively addresses the reviewers' feedback. We appreciate your dedication to advancing the field and look forward to reviewing the revised version of your work.

Best regards

[Note: HTML markup is below. Please do not edit.]

Reviewers' comments:

Reviewer's Responses to Questions

**Comments to the Author**

1. Is the manuscript technically sound, and do the data support the conclusions?

The manuscript must describe a technically sound piece of scientific research with data that supports the conclusions. Experiments must have been conducted rigorously, with appropriate controls, replication, and sample sizes. The conclusions must be drawn appropriately based on the data presented. 

Reviewer #1: Yes

Reviewer #2: Partly

**********

2. Has the statistical analysis been performed appropriately and rigorously? 

Reviewer #1: Yes

Reviewer #2: Yes

**********

3. Have the authors made all data underlying the findings in their manuscript fully available?

The PLOS Data policy requires authors to make all data underlying the findings described in their manuscript fully available without restriction, with rare exception (please refer to the Data Availability Statement in the manuscript PDF file). The data should be provided as part of the manuscript or its supporting information, or deposited to a public repository. For example, in addition to summary statistics, the data points behind means, medians and variance measures should be available. If there are restrictions on publicly sharing data—e.g. participant privacy or use of data from a third party—those must be specified.

Reviewer #1: Yes

Reviewer #2: Yes

**********

4. Is the manuscript presented in an intelligible fashion and written in standard English?

PLOS ONE does not copyedit accepted manuscripts, so the language in submitted articles must be clear, correct, and unambiguous. Any typographical or grammatical errors should be corrected at revision, so please note any specific errors here.

Reviewer #1: Yes

Reviewer #2: Yes

**********

5. Review Comments to the Author

Please use the space provided to explain your answers to the questions above. You may also include additional comments for the author, including concerns about dual publication, research ethics, or publication ethics. (Please upload your review as an attachment if it exceeds 20,000 characters)

Reviewer #1: COMMENTS

Among the events that disrupt sleep health in the world, snoring and sleep breathing disorders are common and well known. I find the research review necessary and important. My suggestions and comments to the authors explaining the "relationship between snoring and sleep stages" in adults and children in their articles are as follows:

Comment 1: Presenting the "percentage of snoring time in TST" values in a table makes it easy to see each value (for the Results section).

Comment 2: For the discussion section, innovative diagnosis and treatments should be presented, apart from the classical treatment of snoring Treatment with an oral appliance. Recently, snoring sound analyzes have been performed meticulously and successfully with machine learning methods applied to polysomnographic measurements (1). In this way, the effects of snoring sound on brain waves (ElectroEncephaloGram, EEG (2), respiration and heart (ElectroCardioGram)) are revealed by examining electrophysiological signal recordings in PSG. Again, by developing sensory and digital devices with computer-based learning methods, diagnosis and treatment of snoring and sleep breathing disorders is done (3).

1. M. Kayabekir, M. Yağanoğlu, C. Köse (2022). SNOROSALAB: A Method Facilitating the Diagnosis of Sleep Breathing Disorders Before Polysomnography, IRBM, Volume 43, Issue 4,

Pages 259-271, ISSN 1959-0318, https://doi.org/10.1016/j.irbm.2021.08.002.

(https://www.sciencedirect.com/science/article/pii/S1959031821000993)

2. Kayabekir, M., & Yağanoğlu, M. (2022). The relationship between snoring sounds and EEG signals on polysomnography. Sleep & breathing = Schlaf & Atmung, 26(3), 1219–1226. https://doi.org/10.1007/s11325-021-02516-8

3. Yağanoğlu, M., Kayabekir, M., & Köse, C. (2017). SNORAP: A Device for the Correction of Impaired Sleep Health by Using Tactile Stimulation for Individuals with Mild and Moderate Sleep Disordered Breathing. Sensors (Basel, Switzerland), 17(9), 2006. https://doi.org/10.3390/s17092006

Comment 3: For the study limitations section: The position of the snorer during sleep in NREM and REM sleep processes is very important in terms of reducing and treating snoring. Correct position of the person during sleep in bed (left or right side, less on his back); It improves NREM and REM sleep durations (3, 4). The results of this study do not provide information about the relationship between snoring and its position, especially in snoring women under the age of 40 and children aged 7 years.

4. Kayabekir M. (2018). Int J Respir Pulm Med, 5:096 Volume 5 | Issue 2 DOI: 10.23937/2378-3516/1410096

Reviewer #2: A manuscript with important findings regarding NREM slow-wave sleep impact on OSA. One important key factor in any respiratory event (including snoring) in sleep apnea is body position. This manuscript does not include any information about body position. Adding this information can affect results and conclusions, therefore must be considered. I suggest some references to the manuscript discussion: Body posture as a factor determining sleep quality in patients using non-invasive ventilation https://pubmed.ncbi.nlm.nih.gov/36973594/

Effect of Sleeping Position on Upper Airway Patency in Obstructive Sleep Apnea Is Determined by the Pharyngeal Structure Causing Collapse https://doi.org/10.1093/sleep/34.4.541

**********

6. PLOS authors have the option to publish the peer review history of their article (what does this mean?). If published, this will include your full peer review and any attached files.

If you choose “no”, your identity will remain anonymous but your review may still be made public.

**Do you want your identity to be public for this peer review?** For information about this choice, including consent withdrawal, please see our Privacy Policy.

Reviewer #1: **Yes: **Dr. Murat Kayabekir

Reviewer #2: **Yes: **Daniel Alfaiate

**********

[NOTE: If reviewer comments were submitted as an attachment file, they will be attached to this email and accessible via the submission site. Please log into your account, locate the manuscript record, and check for the action link "View Attachments". If this link does not appear, there are no attachment files.]

While revising your submission, please upload your figure files to the Preflight Analysis and Conversion Engine (PACE) digital diagnostic tool, https://pacev2.apexcovantage.com/. PACE helps ensure that figures meet PLOS requirements. To use PACE, you must first register as a user. Registration is free. Then, login and navigate to the UPLOAD tab, where you will find detailed instructions on how to use the tool. If you encounter any issues or have any questions when using PACE, please email PLOS at figures@plos.org. Please note that Supporting Information files do not need this step.

# Author comment: Author response to Decision Letter 0

**DOI:** 10.1371/journal.pone.0295232.r002

2 Aug 2023

Reviewer #1:

 We would like to thank the reviewer for the latest review and we have answered each of the points below. The paper has been checked by a native English-speaking medical editor. We hope that this more substantial reworking of the manuscript is satisfactory.

Among the events that disrupt sleep health in the world, snoring and sleep breathing disorders are common and well known. I find the research review necessary and important. My suggestions and comments to the authors explaining the "relationship between snoring and sleep stages" in adults and children in their articles are as follows:

Comment 1: Presenting the "percentage of snoring time in TST" values in a table makes it easy to see each value (for the Results section).

Thank you for pointing this out. For clarity, all numbers are presented in numerical form. See figures.

Comment 2: For the discussion section, innovative diagnosis and treatments should be presented, apart from the classical treatment of snoring Treatment with an oral appliance. Recently, snoring sound analyzes have been performed meticulously and successfully with machine learning methods applied to polysomnographic measurements (1). In this way, the effects of snoring sound on brain waves (ElectroEncephaloGram, EEG (2), respiration and heart (ElectroCardioGram)) are revealed by examining electrophysiological signal recordings in PSG. Again, by developing sensory and digital devices with computer-based learning methods, diagnosis and treatment of snoring and sleep breathing disorders is done (3).

Thank you for pointing this out. We have added this limitation with references to the Discussion. 

Comment 3: For the study limitations section: The position of the snorer during sleep in NREM and REM sleep processes is very important in terms of reducing and treating snoring. Correct position of the person during sleep in bed (left or right side, less on his back); It improves NREM and REM sleep durations (3, 4). The results of this study do not provide information about the relationship between snoring and its position, especially in snoring women under the age of 40 and children aged 7 years.

Thank you very much for pointing this out. We compared the percentage of snoring time between the supine and non-supine (i.e., left and right lateral, and prone) positions. 

In adult patients (n = 93), there were no significant differences in the percentage of snoring time between the supine and non-supine positions in any sleep stage (Fig. 4a). However, in children (n = 47), the percentage of snoring time in the non-supine position was significantly higher during N3 sleep (p = 0.046) and significantly lower during N1 sleep (p = 0.043) compared with the supine position (Fig. 4b). A similar tendency was also seen in adult women aged < 40 years (n = 33) but the difference was not significant.

We have added these findings to the Results section and figures, and we have revised the discussion accordingly.

Reviewer #2: 

We would like to thank the reviewer for the latest review and we have answered each of the points below. The paper has been checked by a native English-speaking medical editor. We hope that this more substantial reworking of the manuscript is satisfactory.

A manuscript with important findings regarding NREM slow-wave sleep impact on OSA. One important key factor in any respiratory event (including snoring) in sleep apnea is body position. This manuscript does not include any information about body position. Adding this information can affect results and conclusions, therefore must be considered.

 Thank you very much for pointing this out. We compared the percentage of snoring time between the supine and non-supine (i.e., left and right lateral, and prone) positions. 

In adult patients (n = 93), there were no significant differences in the percentage of snoring time between the supine and non-supine positions in any sleep stage (Fig. 4a). However, in children (n = 47), the percentage of snoring time in the non-supine position was significantly higher during N3 sleep (p = 0.046) and significantly lower during N1 sleep (p = 0.043) compared with the supine position (Fig. 4b). A similar tendency was also seen in adult women aged < 40 years (n = 33) but the difference was not significant.

We have added these findings to the Results section and figures, and we have revised the discussion accordingly.

Attachment Submitted filename: Response to Reviewers PLOS ONE.docx Click here for additional data file.

# Peer review: Decision Letter 1

**DOI:** 10.1371/journal.pone.0295232.r003

**Authors:** Marco Zaffanello

8 Nov 2023

PONE-D-23-01986R1During which sleep stages does isolated snoring occur in children and adults?PLOS ONE

Dear Dr. Suzuki,

Thank you for submitting your manuscript to PLOS ONE. After careful consideration, we feel that it has merit but does not fully meet PLOS ONE’s publication criteria as it currently stands. Therefore, we invite you to submit a revised version of the manuscript that addresses the points raised during the review process.

Please submit your revised manuscript by Dec 23 2023 11:59PM. If you will need more time than this to complete your revisions, please reply to this message or contact the journal office at plosone@plos.org. When you're ready to submit your revision, log on to https://www.editorialmanager.com/pone/ and select the 'Submissions Needing Revision' folder to locate your manuscript file.

Please include the following items when submitting your revised manuscript:

- A rebuttal letter that responds to each point raised by the academic editor and reviewer(s). You should upload this letter as a separate file labeled 'Response to Reviewers'.
- A marked-up copy of your manuscript that highlights changes made to the original version. You should upload this as a separate file labeled 'Revised Manuscript with Track Changes'.
- An unmarked version of your revised paper without tracked changes. You should upload this as a separate file labeled 'Manuscript'.

If you would like to make changes to your financial disclosure, please include your updated statement in your cover letter. Guidelines for resubmitting your figure files are available below the reviewer comments at the end of this letter.

If applicable, we recommend that you deposit your laboratory protocols in protocols.io to enhance the reproducibility of your results. Protocols.io assigns your protocol its own identifier (DOI) so that it can be cited independently in the future. For instructions see: https://journals.plos.org/plosone/s/submission-guidelines#loc-laboratory-protocols. Additionally, PLOS ONE offers an option for publishing peer-reviewed Lab Protocol articles, which describe protocols hosted on protocols.io. Read more information on sharing protocols at https://plos.org/protocols?utm_medium=editorial-email&utm_source=authorletters&utm_campaign=protocols.

We look forward to receiving your revised manuscript.

Kind regards,

Marco Zaffanello

Academic Editor

PLOS ONE

Journal Requirements:

Please review your reference list to ensure that it is complete and correct. If you have cited papers that have been retracted, please include the rationale for doing so in the manuscript text, or remove these references and replace them with relevant current references. Any changes to the reference list should be mentioned in the rebuttal letter that accompanies your revised manuscript. If you need to cite a retracted article, indicate the article’s retracted status in the References list and also include a citation and full reference for the retraction notice.

Additional Editor Comments (if provided):

This study appears to address several issues related to snoring, sleep stages, and obstructive sleep apnea (OSA).

The study's title mentions "isolated snoring," but much of the content focuses on snoring in patients with OSA and during different sleep stages. This discrepancy can confuse readers and may not fully reflect the actual scope of the study. the title could be more accurate and representative. For instance, "Exploring the Dynamics of Snoring in Relation to Sleep Stages and Obstructive Sleep Apnea: Implications for Gender Differences and Upper Airway Collapsibility" might be a more suitable option. However, this is not strictly necessary..

The study highlights that snoring is more predominant during N3 and N2 sleep stages and less present during REM sleep. These findings appear to align with some of the existing knowledge about sleep physiology and snoring. However, the discussion in the first passage and the description of the study results need to be more accurately presented. In the discussion, it is stated that snoring is predominant during deep sleep (N3) and less present during REM sleep, while the study suggests that snoring was less predominant during N3 and N2 sleep stages and more present during REM sleep. However, the discussion seems to contradict these results, creating confusion.

The study identifies significant gender differences in the percentages of snoring time, particularly during N3 sleep. This observation can have important implications for understanding the dynamics of OSA and gender-related sleep disorders.

The analysis of upper airway collapsibility and its relationship with snoring are relevant aspects of the study. The discovery that this collapsibility is greater in men after controlling for factors like BMI, age, and the severity of apnea adds an additional layer of understanding to the cause of snoring in patients with OSA.

The investigation into the effects of oral appliance (OA) treatment on snoring is intriguing. The significant reduction in the apnea-hypopnea index (AHI) and changes in sleep stages after treatment indicate that OA may be effective in improving sleep-related breathing disorders.

In summary, the study provides valuable insights into the relationship between snoring, sleep stages, and OSA, despite some discrepancies between the title and the content. However, for greater clarity, it would be helpful to improve the consistency between the discussion and the reported results, as well as to consider a title more suited to the actual scope of the research.

[Note: HTML markup is below. Please do not edit.]

Reviewers' comments:

Reviewer's Responses to Questions

**Comments to the Author**

1. If the authors have adequately addressed your comments raised in a previous round of review and you feel that this manuscript is now acceptable for publication, you may indicate that here to bypass the “Comments to the Author” section, enter your conflict of interest statement in the “Confidential to Editor” section, and submit your "Accept" recommendation.

Reviewer #2: All comments have been addressed

**********

2. Is the manuscript technically sound, and do the data support the conclusions?

The manuscript must describe a technically sound piece of scientific research with data that supports the conclusions. Experiments must have been conducted rigorously, with appropriate controls, replication, and sample sizes. The conclusions must be drawn appropriately based on the data presented. 

Reviewer #2: Yes

**********

3. Has the statistical analysis been performed appropriately and rigorously? 

Reviewer #2: Yes

**********

4. Have the authors made all data underlying the findings in their manuscript fully available?

The PLOS Data policy requires authors to make all data underlying the findings described in their manuscript fully available without restriction, with rare exception (please refer to the Data Availability Statement in the manuscript PDF file). The data should be provided as part of the manuscript or its supporting information, or deposited to a public repository. For example, in addition to summary statistics, the data points behind means, medians and variance measures should be available. If there are restrictions on publicly sharing data—e.g. participant privacy or use of data from a third party—those must be specified.

Reviewer #2: Yes

**********

5. Is the manuscript presented in an intelligible fashion and written in standard English?

PLOS ONE does not copyedit accepted manuscripts, so the language in submitted articles must be clear, correct, and unambiguous. Any typographical or grammatical errors should be corrected at revision, so please note any specific errors here.

Reviewer #2: Yes

**********

6. Review Comments to the Author

Please use the space provided to explain your answers to the questions above. You may also include additional comments for the author, including concerns about dual publication, research ethics, or publication ethics. (Please upload your review as an attachment if it exceeds 20,000 characters)

Reviewer #2: The authors have adequately addressed comments raised in a previous round of review therefore this manuscript is now acceptable for publication

**********

7. PLOS authors have the option to publish the peer review history of their article (what does this mean?). If published, this will include your full peer review and any attached files.

If you choose “no”, your identity will remain anonymous but your review may still be made public.

**Do you want your identity to be public for this peer review?** For information about this choice, including consent withdrawal, please see our Privacy Policy.

Reviewer #2: **Yes: **Daniel Alfaiate

**********

[NOTE: If reviewer comments were submitted as an attachment file, they will be attached to this email and accessible via the submission site. Please log into your account, locate the manuscript record, and check for the action link "View Attachments". If this link does not appear, there are no attachment files.]

While revising your submission, please upload your figure files to the Preflight Analysis and Conversion Engine (PACE) digital diagnostic tool, https://pacev2.apexcovantage.com/. PACE helps ensure that figures meet PLOS requirements. To use PACE, you must first register as a user. Registration is free. Then, login and navigate to the UPLOAD tab, where you will find detailed instructions on how to use the tool. If you encounter any issues or have any questions when using PACE, please email PLOS at figures@plos.org. Please note that Supporting Information files do not need this step.

# Author comment: Author response to Decision Letter 1

**DOI:** 10.1371/journal.pone.0295232.r004

15 Nov 2023

We would like to thank the reviewer for the latest review and we have answered each of the points below. We review the reference list to ensure that it is complete and correct. We hope that this more substantial reworking of the manuscript is satisfactory.

Additional Editor Comments

This study appears to address several issues related to snoring, sleep stages, and obstructive sleep apnea (OSA).

The study's title mentions "isolated snoring," but much of the content focuses on snoring in patients with OSA and during different sleep stages. This discrepancy can confuse readers and may not fully reflect the actual scope of the study. the title could be more accurate and representative. For instance, "Exploring the Dynamics of Snoring in Relation to Sleep Stages and Obstructive Sleep Apnea: Implications for Gender Differences and Upper Airway Collapsibility" might be a more suitable option. However, this is not strictly necessary..

Thank you for your wonderful suggestion. We agree and have changed the title to “Exploring the Dynamics of Snoring in Relation to Sleep Stages: Implications for Gender Differences, Sleep Position, and Upper Airway Collapsibility”. Since we analyzed the percentage of snoring time of non-OSA women, we do not want to include the word "OSA" in the title.

The study highlights that snoring is more predominant during N3 and N2 sleep stages and less present during REM sleep. These findings appear to align with some of the existing knowledge about sleep physiology and snoring. However, the discussion in the first passage and the description of the study results need to be more accurately presented. In the discussion, it is stated that snoring is predominant during deep sleep (N3) and less present during REM sleep, while the study suggests that snoring(respiratory events?) was less predominant during N3 and N2 sleep stages and more present during REM sleep. However, the discussion seems to contradict these results, creating confusion.

Thank you for pointing this out. Words and sentences were deleted and added to maintain consistency in the Discussion section.

Attachment Submitted filename: renamed_359ed.docx Click here for additional data file.

# Editor report: Decision Letter 2

**DOI:** 10.1371/journal.pone.0295232.r005

**Authors:** Marco Zaffanello

20 Nov 2023

Exploring the Dynamics of Snoring in Relation to Sleep Stages: Implications for Gender Differences, Sleep Position, and Upper Airway Collapsibility.

PONE-D-23-01986R2

Dear Dr. Suzuki,

We’re pleased to inform you that your manuscript has been judged scientifically suitable for publication and will be formally accepted for publication once it meets all outstanding technical requirements.

Within one week, you’ll receive an e-mail detailing the required amendments. When these have been addressed, you’ll receive a formal acceptance letter and your manuscript will be scheduled for publication.

An invoice for payment will follow shortly after the formal acceptance. To ensure an efficient process, please log into Editorial Manager at http://www.editorialmanager.com/pone/, click the 'Update My Information' link at the top of the page, and double check that your user information is up-to-date. If you have any billing related questions, please contact our Author Billing department directly at authorbilling@plos.org.

If your institution or institutions have a press office, please notify them about your upcoming paper to help maximize its impact. If they’ll be preparing press materials, please inform our press team as soon as possible -- no later than 48 hours after receiving the formal acceptance. Your manuscript will remain under strict press embargo until 2 pm Eastern Time on the date of publication. For more information, please contact onepress@plos.org.

Kind regards,

Marco Zaffanello

Academic Editor

PLOS ONE

Additional Editor Comments (optional):

All comments have been addressed

Reviewers' comments:

# Editor report: Acceptance letter

**DOI:** 10.1371/journal.pone.0295232.r006

**Authors:** Marco Zaffanello

8 Jan 2024

PONE-D-23-01986R2 

PLOS ONE

Dear Dr. Suzuki, 

I'm pleased to inform you that your manuscript has been deemed suitable for publication in PLOS ONE. Congratulations! Your manuscript is now being handed over to our production team.

At this stage, our production department will prepare your paper for publication. This includes ensuring the following:

* All references, tables, and figures are properly cited

* All relevant supporting information is included in the manuscript submission,

* There are no issues that prevent the paper from being properly typeset

If revisions are needed, the production department will contact you directly to resolve them. If no revisions are needed, you will receive an email when the publication date has been set. At this time, we do not offer pre-publication proofs to authors during production of the accepted work. Please keep in mind that we are working through a large volume of accepted articles, so please give us a few weeks to review your paper and let you know the next and final steps. 

Lastly, if your institution or institutions have a press office, please let them know about your upcoming paper now to help maximize its impact. If they'll be preparing press materials, please inform our press team within the next 48 hours. Your manuscript will remain under strict press embargo until 2 pm Eastern Time on the date of publication. For more information, please contact onepress@plos.org.

If we can help with anything else, please email us at customercare@plos.org.

Thank you for submitting your work to PLOS ONE and supporting open access. 

Kind regards, 

PLOS ONE Editorial Office Staff

on behalf of

Prof. Marco Zaffanello 

Academic Editor

PLOS ONE
